# ALK rearrangement-associated renal cell carcinoma morphologically mimicking mucinous tubular and spindle cell carcinoma: a case report

**DOI:** 10.1186/s13000-022-01238-z

**Published:** 2022-06-19

**Authors:** Keita Kai, Shohei Tobu, Shinichi Kido, Shuji Mikami, Kengo Takeuchi, Akito Dobashi, Yuki Togashi, Mitsuru Noguchi, Shinichi Aishima

**Affiliations:** 1 Department of Pathology, Saga University Hospital, Nabeshima 5-1-1, Saga, 849-8501 Japan; 2 Department of Urology, Saga University Faculty of Medicine, Saga, Japan; 3 Department of Pathology & Microbiology, Saga University Faculty of Medicine, Saga, Japan; 4 Department of Pathology, National Hospital Organization Saitama Hospital, Saitama, Japan; 5 Division of Pathology, The Cancer Institute, Japanese Foundation for Cancer Research, Tokyo, Japan; 6 Department of Pathology, The Cancer Institute Hospital, Japanese Foundation for Cancer Research, Tokyo, Japan; 7 Pathology Project for Molecular Targets, The Cancer Institute, Japanese Foundation for Cancer Research, Tokyo, Japan

**Keywords:** Renal cell carcinoma, Anaplastic lymphoma kinase, Mucinous tubular and spindle cell carcinoma, Case report

## Abstract

**Background:**

Anaplastic lymphoma kinase rearrangement-associated renal cell carcinoma (ALK-RCC) is an extremely rare tumor and ALK-RCC that mimics mucinous tubular and spindle cell carcinoma (MTSCC) has been very reported only in one instance.

**Case presentation:**

A 42-year-old Japanese woman was admitted to our hospital for the treatment of a left renal tumor measuring 5 cm in maximum dimension. She underwent a laparoscopic left nephrectomy. Histologically, the tumor formed tubular or focally papillary structures with a small amount of spindle-shaped tumor cells against the background of prominent extracellular mucin. Although the tumor cells were negative for immunohistochemistry (IHC) of alpha-methylacyl-CoA racemase (AMACR) and lymph node metastasis was presented (these are atypical findings for MTSCC), we initially diagnosed the tumor as MTSCC based on its morphological characteristics with mucin deposition. However, an additional IHC analysis revealed that the tumor cells were diffusely positive for ALK-IHC. In addition, TPM3 exon 8 – ALK exon 20 fusion gene was detected by RNA sequencing. The tumor was thus correctly diagnosed as ALK rearrangement-associated renal cell carcinoma (ALK-RCC).

**Conclusions:**

Since the use of molecular targeted therapy with an ALK inhibitor for ALK-RCC is promising, the correct pathological diagnosis of ALK-RCC is quite important. We strongly recommend that ALK-IHC be routinely performed for renal tumors with negative AMACR staining that mimic MTSCC.

## Background

Anaplastic lymphoma kinase rearrangement-associated renal cell carcinoma (ALK-RCC) is a very rare tumor that accounts for <1% of all renal neoplasms [1]. Although ALK-RCC is currently considered an "emerging/provisional" type of renal cell carcinoma in the 2016 World Health Organization classification [2], the Genitourinary Pathology Society recently proposed categorizing ALK-RCC as a "novel entity" based on an accumulation of studies [3]. Because of its heterogeneous and diverse morphologies, the differential diagnosis of ALK-RCC varies widely, involving renal medullary carcinoma, collecting duct carcinoma, papillary RCC, MiT family translocation RCC, clear cell RCC with rhabdoid features, metanephric adenoma, thyroid-like follicular RCC, and mucinous tubular and spindle-cell carcinoma (MTSCC) [4].

We present a didactic case of ALK-RCC which was initially diagnosed as MTSCC. This case taught us the importance of considering the possibility of ALK-RCC in the pathological diagnosis of unusual renal tumors.

## Case presentation

### Clinical summary

A 42-year-old Japanese woman was admitted to our hospital for the treatment of a left renal tumor that had been detected by a medical checkup. She was neither a habitual drinker nor a smoker. She had no remarkable medical or family history. The physical examination on admission was unremarkable. Laboratory hematologic, serological, and coagulation tests on admission revealed no abnormalities. Abdominal computed tomography and magnetic resonance imaging revealed a well-circumscribed solid tumor measuring 5 × 4 cm in diameter at the left kidney. The tumor was hypovascular and protruded toward the renal pelvis. Radiologically, papillary RCC or chromophobe RCC were considered as differential diagnoses. A needle biopsy was performed, and the pathological report of biopsy specimens was "unclassified mucin-producing renal cell carcinoma."

The patient underwent a laparoscopic left nephrectomy. Intraoperatively, one enlarged lymph node was identified at the hilum of the left kidney, and it was surgically dissected. The postoperative course was uneventful, and the patient was discharged from the hospital on the sixth postoperative day. The final stage based on TNM staging system was Stage III (pT1bN1M0). She was periodically followed without adjuvant therapy, and no sign of recurrence had been detected at the time of this writing (24 months after the surgery).

### Pathological findings of resected specimens

**Fig. 1:**
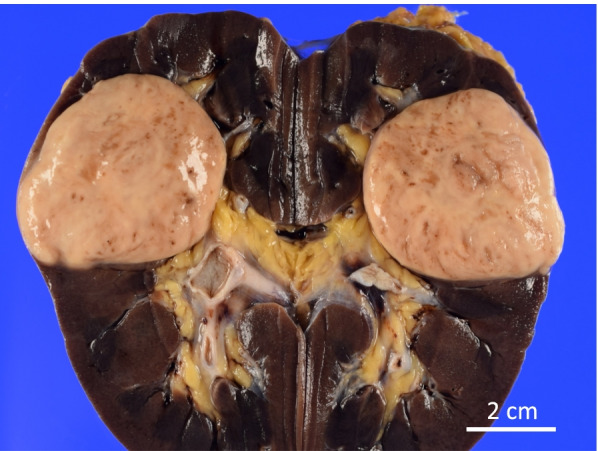
Gross appearance of the tumor. The tumor was well-circumscribed, tan-white to tan-pink and homogeneous, and solid, and it showed expansile growth

**Fig. 2:**
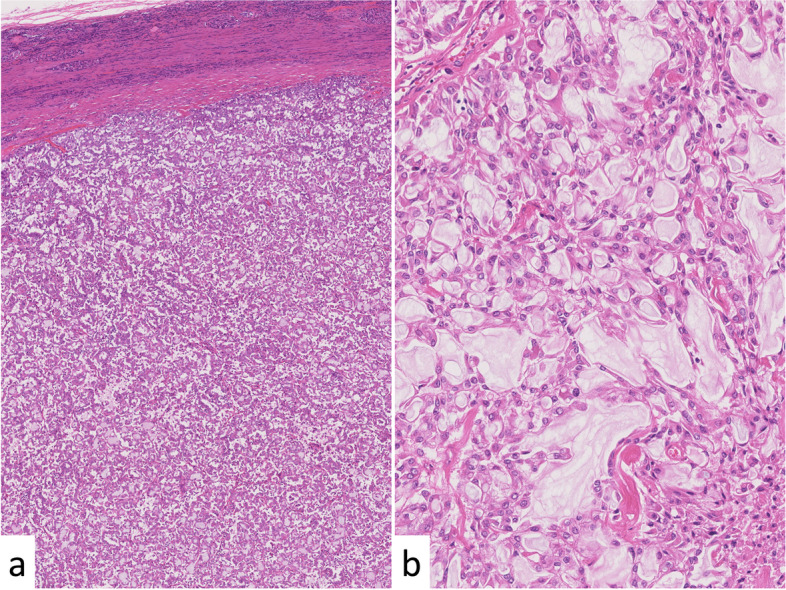
Histological appearance. **a**: Low magnification (×50). The tumor showed expansive growth and formed tubular or papillary structures. **b**: High magnification (×200). The tumor cells had eosinophilic cytoplasm and small-round nuclei. A small amount of spindle-shaped tumor cells and prominent extracellular mucin were observed

**Fig. 3:**
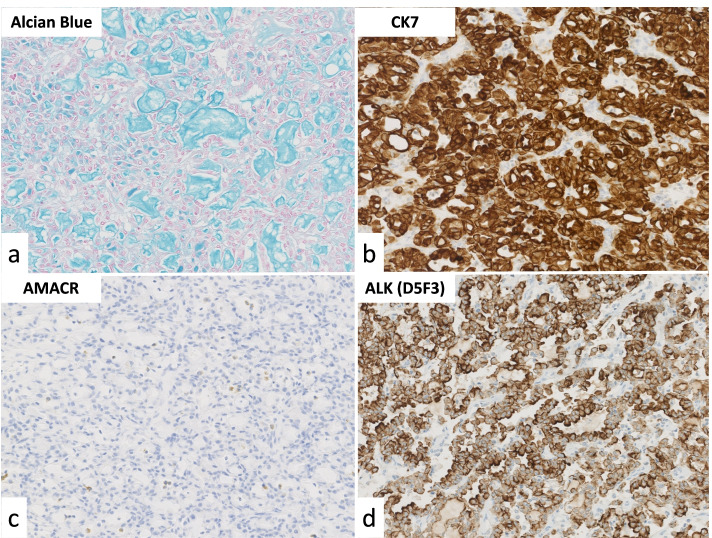
**a** Extracellular mucin deposition is highlighted by alcian blue staining). IHC results for CK7 **(b)**, AMACR **(c)**, and ALK(D5F3) **(d**). The magnification of all four slides is ×200. The tumor showed diffuse positivity at the cytoplasm and diffuse and strong positivity at the cell membrane

The cut section of the tumor (Fig. 1) revealed a well-circumscribed tan-white to tan-pink and homogeneous solid tumor showing expansile growth at upper pole to interpolar region of the left kidney. Histologically, tubular or focally papillary structures compose3d of tumor cells with eosinophilic cytoplasm and small-round nuclei were observed, and a small amount of spindle-shaped tumor cells and its transition figures were intermingled (Fig. 2). The WHO/ISUP grade was Grade 2. Prominent extracellular mucin deposition highlighted by alcian blue staining was observed (Fig. 3a).

Immunohistochemically, the tumor cells were positive for cytokeratin (CK) 7 (clone OV-TL 12/30, ready to use; Agilent Technologies, Santa Clara, CA) (Fig. 3b), paired box gene (PAX) 8 (clone MRQ-50, ready to use; Roche, Mannheim, Germany), and vimentin but negative for alpha-methylacyl-CoA racemase (AMACR) (clone 13H4, ready to use; Agilent Technologies) (Fig. 3c) and CD10 (clone SP67, ready to use; Roche). Lymph node metastasis was observed in one of the dissected lymph nodes. Although the lymph node metastasis and the negativity of AMACR are not typical findings of MTSCC, we initially diagnosed this tumor as MTSCC based on its morphological characteristics with mucin deposition.

We presented this case as an MTSCC at the 380th Kyushu-Okinawa slide conference (March 2021) held by the Kyushu-Okinawa Division of Japanese Society of Pathology. At that conference, some participants pointed out the possibility of the tumor being ALK-RCC. Following their comments, we performed an additional immunohistochemistry (IHC) analysis of the tumor by using ALK (D5F3) (clone D5F3, ready to use; Roche), and the tumor showed diffuse cytoplasmic and diffuse strong membranous positivity (Fig. 3d). In addition, TPM3 exon 8 – ALK exon 20 fusion gene was detected by RNA sequencing. We thus finally reached the correct diagnosis of ALK-RCC.

## Discussion and conclusions

RCC with rearrangement of ALK gene was first described by Debelenko et al. in 2011 [5]. Their report described a pediatric case with sickle cell trait, and the fusion partner of ALK gene was VCL gene. The first adult cases of ALK-RCC without a genetic background were reported in 2012 by Sugawara et al. [6]. They screened 355 RCC cases by ALK-IHC (using an intercalated antibody-enhanced polymer method) and identified two ALK-RCC cases which were confirmed to have TPM3-ALK and EML4-ALK fusion by fluorescence *in situ* hybridization assays.

Although ALK-RCC is extremely rare, its pathological features have been gradually revealed by the accumulation of case reports and studies [7]. ALK-RCC usually macroscopically presents a well- or ill-demarcated solid tumor, but cystic changes or a cystic lesion have also been reported [4, 8, 9]. The histological morphology of ALK-RCC is highly variable and heterogeneous, not only between cases, but also within individual cases [4]. Most ALK-RCC cases have exhibited mixed architectural patterns, including papillary, tubular, trabecular, tubulocystic, and solid patterns. The tumor cells typically showed eosinophilic cytoplasm and various degrees of mucin deposition [4, 7].

ALK-RCCs usually express PAX 8, CK7, and vimentin, and no specific IHC marker other than ALK-IHC has been reported [4]. It has been suggested that a strong membranous stain is characteristic of TPM3-ALK fusion [6], and our patient's case also showed strong membranous positivity for ALK-IHC, supporting that suggestion. The reported fusion partners to ALK gene include VCL, TPM3, EML4, HOOK1, and STRN gene [7]. Recently, PLEKHA7, CLIP1, KIF5B and KIAA1217 were reported as fusion partners of ALK gene [4, 10].

As described in the Introduction, the differential diagnosis of ALK-RCC varies widely, and ALK-RCC may mimic various renal tumors. As ALK-RCC may show mucin deposition and a tubular structure, the importance of the differential diagnosis of MTSCC has been noted [7]. However, to the best of our knowledge, no single case report of ALK-RCC that mimics MTSCC has been reported. Only one multi-institutional study of 12 cases described an ALK-RCC case morphologically resembling MTSCC which showed immunoreactivity compatible with MTSCC, i.e., positivity for PAX8, CK7, CD10, AMACR, and vimentin [4].

MTSCC is also a relatively rare epithelial neoplasm of low malignant potential with characteristic histologic features [11]. Although it was originally described as a tumor arising from cells of the loop of Henle or the collecting duct, the expression of CK7 and AMACR suggested its proximal nephron origin and its close resemblance to papillary RCC [12]. In the present case, although the findings of a lack of AMACR expression and lymph node metastasis were not typical findings of MTSCC, we initially considered MTSCC based on reports of rare cases of MTSCC that lacked AMACR expression and presented with lymph node metastasis, and distant metastasis [10, 13]. Considering the above-mentioned ALK-RCC case morphologically resembling MTSCC with AMACR expression, we strongly recommend an analysis of ALK expression by IHC when diagnosing a renal tumor that mimics MTSCC.

In conclusion, we have described a case of ALK-RCC which morphologically mimicked MTSCC. Since the use of molecular targeted therapy with an ALK inhibitor for cases of ALK-RCC is promising [6], the correct pathological diagnosis of ALK-RCC is quite important. In light of the lesson learned in the present case, we strongly recommend the routine performance of ALK-IHC in the pathological diagnosis of MTSCC.

## Data Availability

Data are available on reasonable request from the corresponding author due to privacy or other restrictions.

## Abbreviations

ALK-RCC: Anaplastic lymphoma kinase rearrangement-associated renal cell carcinoma
MTSCC: Mucinous tubular and spindle-cell carcinoma
CK: Cytokeratin
PAX 8: Paired box gene 8
AMACR: Alpha-methylacyl-CoA racemase
IHC: Immunohistochemistry
